# Supplementary material for: Predicting visual function by interpreting a neuronal wiring diagram
Source: Nature. 2024 Oct 2;634(8032):113–23. doi: 10.1038/s41586-024-07953-5 (PMC11446822; doi:10.1038/s41586-024-07953-5)
Supplement: Supplementary file 6 — CRF and ERF predictions for individual TmY4 and TmY9 cells. Analogous to Supplementary Data 3, but for TmY target types. Shown are the top four monosynaptic pathways, the strongest pathway passing through each of the top ten intermediary types (ranking from Extended Data Fig. 7), and the trisynaptic pathway Tm1–TmY–Dm3–TmY (see the section entitled Prediction of spatial normalization). [file 41586_2024_7953_MOESM6_ESM.zip › DataS4/TmY9qΓèÑ/720575940638909493.pdf]

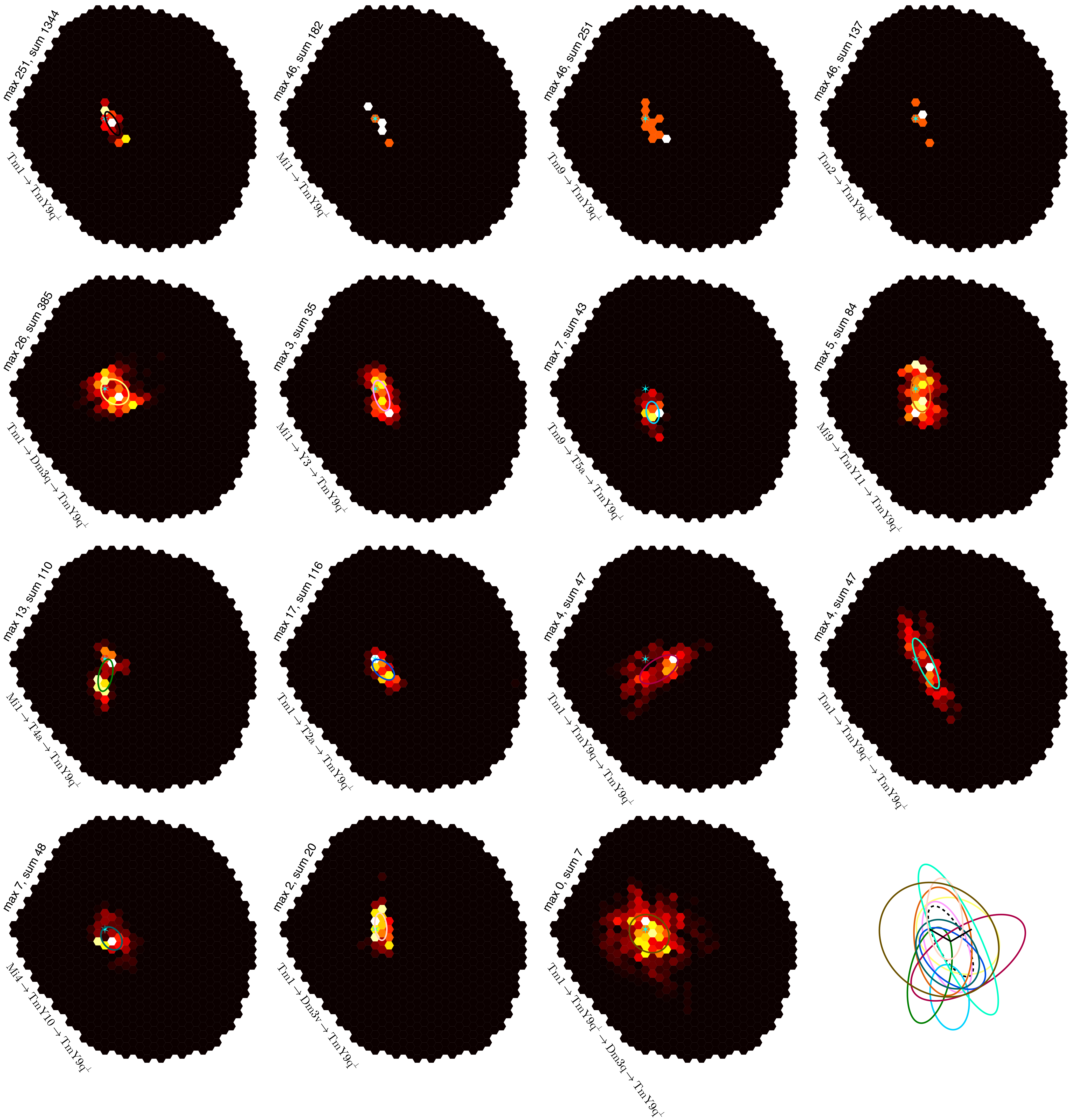
max 251, sum 1344
Tm1 → TmY9q⊥
max 46, sum 182
Mi1 → TmY9q⊥
max 46, sum 251
Tm9 → TmY9q⊥
max 46, sum 137
Tm2 → TmY9q⊥
max 26, sum 385
Tm1 → Dm3q → TmY9q⊥
max 3, sum 35
Mi1 → Y3 → TmY9q⊥
max 7, sum 43
Tm9 → T5a → TmY9q⊥
max 5, sum 84
Mi9 → TmY11 → TmY9q⊥
max 13, sum 110
Mi1 → T4a → TmY9q⊥
max 17, sum 116
Tm1 → T2a → TmY9q⊥
max 4, sum 47
Tm1 → TmY9q → TmY9q⊥
max 4, sum 47
Tm1 → TmY9q⊥ → TmY9q⊥
max 7, sum 48
Mi4 → TmY10 → TmY9q⊥
max 2, sum 20
Tm1 → Dm3v → TmY9q⊥
max 0, sum 7
Tm1 → TmY9q⊥ → Dm3q → TmY9q⊥
